# Tactical Knowledge by Decision Making and Motor Efficiency of Young Football Players in Different Playing Positions during a Three-a-Side Small-Sided Game

**DOI:** 10.3390/bs13040310

**Published:** 2023-04-05

**Authors:** Rui Matos, Carlos Moreira, Emília Alves, José Eduardo Teixeira, Filipe Rodrigues, Diogo Monteiro, Raul Antunes, Pedro Forte

**Affiliations:** 1 Department of Sport, Higher Institute of Educational Sciences of the Douro, 4560-708 Penafiel, Portugal; 2 Department of Sports Sciences, University of Trás-os-Montes e Alto Douro, 5001-801 Vila Real, Portugal; 3 CI-ISCE, Higher Institute of Educational Sciences of the Douro, 4560-708 Penafiel, Portugal; 4 Research Centre in Physical Activity, Health, and Leisure, CIAFEL, 4200-450 Porto, Portugal; 5 Department of Sport Science, Instituto Politécnico de Bragança, 5300-253 Bragança, Portugal; 6 Department of Sport Sciences, Polytechnic Institute of Guarda, 6300-559 Guarda, Portugal; 7 Research Center in Sports, Health, and Human Development, 6201-001 Covilhã, Portugal; 8 ESECS- Polytechnic of Leiria, 2411-901 Leiria, Portugal; filipe.rodrigues@ipleiria.pt (F.R.);; 9 Life Quality Research Center, 2040-413 Leiria, Portugal

**Keywords:** positional, tactical, decision making, motor efficiency, football

## Abstract

The aim of this study was to compare the tactical knowledge of young football players in different playing positions during a three-a-side small-sided game (SSG). Observational data was collected from 71 players (M = 12.16; SD = 1.55 years): 11 goalkeepers, 22 defenders, 15 midfielders, and 23 forwards. In total, 4 min of three-a-side SSGs (GR + 3 vs. 3 + GR) were recorded to assess tactical performance using a digital camera (GoPro Hero 6^®^ version 02.01). The SSGs were performed on a field with a constant area (36 × 27 m). Video analyses were performed using LongoMatch^®^ version 1.5.9 to record football performance; we then assessed tactical performance by using the Football Tactical Assessment System (Fut-Sat). This instrument evaluates the average of well-defined action indexes for each game in decision making principle and motor skills, specifically: (i) Decision Making Index (DMI); (ii) Motor Effectiveness Index (MEI); (iii) Effectiveness Index (I). The indexes were calculated by the ratio between the correct actions and the total. The Kruskal–Wallis test was used to evaluate differences between playing positions. The results showed that tactical performance by principles seems to be significantly different according to playing position. Differences were found between defenders and forwards (H = −11.92; *p* = 0.03) and defenders and midfielders (H = −16.13; *p* = 0.01) in contention principle. In conclusion, tactical knowledge of training based on the principles of the game can help coaches and players better understand and predict each player’s actions during the game.

## 1. Introduction

Sport practice has been considered essential for encouraging individual development of technical, physical, cognitive, and social skills [1]. Sports training and long-term preparation has been mainly focused on specific skills and behaviors improvement in adolescents [1,2]. Team invasion sports are a particularly significant sporting activity in many societies, attracting practitioners to practice and compete in cooperation with others [3]. Sport practice is a precursor for improving quality of life and motor and cognitive development [4,5]. Sports can be classified as invasion-based if they share the same structure, such as being built of two teams who dispute object possession in a restricted playing area with the objective of scoring in the opponent’s goal and defending their own goal; the team that scored the most goals at the end of time is the winner [6]. Football is a team invasion sport which emphasizes a goal-oriented performance amongst teammates and opponents with a contextual dependency [7,8].

It is fundamental for football players to develop tactical knowledge, which is mediated by decision making and motor efficiency [9,10]. The tactical knowledge seems to be influenced by field position, though it can also be considered that each field position leads to specialized decision making and motor efficiency across the developmental stages [11,12,13]. Specifically, field positions are related to specific tasks to promote dynamic interpersonal coordination during invasion of the opponent’s field, with the aim being to score more points within the time–space limit than the opposing team [14,15]. Thus, the different defensive and offensive game phases define the activities that each player has according to ball possession [16,17]. The offensive phase is characterized by the construction of offensive actions, as well as the creation of finalization situations and the finalization itself [18]. In the defensive phase, players have to prevent the construction of offensive actions, finalization situations, and opponent goal opportunities [19]. Moreover, it is possible to split the game into four moments: offensive transition, offensive organization, defensive transition, and defensive organization [20]. The game’s complexity is dependent on the achievement of team objectives. Thus, a set of options for success must be provided [21]. Player performance is dependent on the tactical abilities and cognitive processes underlying decision making [22,23]. Indeed, previous studies have reported an association between tactical performance, specific motor skills, perceptual–cognitive–motor skills, and decision making effectiveness in athletic development [24,25,26]. Specifically, the small-sided games (SSGs) have contextual dynamics that improve individual and collective technical–tactical expertise in young football players [27,28]. During the game, it can be highlighted that: (i) most of the game’s actions happen without ball; (ii) technical skills may be enhanced or blinded by the tactical understanding or knowledge of each player; (iii) lacking tactical knowledge may limit the execution of technical skills [21]. Even more, individual tactical knowledge can generate diverse actions as a result of divergent thinking among players [29]. Thus, it is possible for each player to choose which action will be the best in a given game situation [30]. 

Improving tactical knowledge and understanding tactical principles has been shown as extremely important to solving game problems and constrains [31,32]. Technical–tactical training in football is understood as a set of game principles such as penetration, offensive coverage, mobility, space, restraint, defensive coverage, balance, and concentration [33]. As players accumulate football experience, greater should be the acquired knowledge about technical–tactical principles. Thus, players may perceive relevant signals more clearly, supporting proper decision making for each game principle in a given situation [31]. Thus, decision making and motor efficiency skills enable the players to use available information to support movement behaviors using specific skills. Developing constrained training tasks is a commonly used strategy to improve tactical knowledge. SSGs, also known as conditioned games, are constrained game-based drills that change the structural dynamic of the match [34,35]. SSGs can be used to improve tactical knowledge [36]. Activities based on reduced exercises are part of training task design used in team sports [37]. Among other games, SSGs are reduced games that alter the structural dynamics of formal games [38]. Tactical knowledge can be assessed by declarative and/or procedural knowledge [39]. Tactical knowledge refers to the ability to know what to do in a given game situation [40]. This knowledge is recurrently assessed using player response verbalization [30]. Tactical procedural knowledge is related to the execution of a motor action, adapting the technical gesture to a game situation [36,39]. Tactical procedural knowledge has been assessed using field tests, such as the FUT-SAT tactical assessment system, which is characterized by an assessment in a SSG over a given period of time [32,41]. Similar to the FUT-SAT, the tactical performance assessment tool of Costa et al. [41] allows the assessment of motor effectiveness for each game principle.

The importance of the assessment of tactical knowledge and involving variables is a determinant of success in individual and collective performance for coaches and players [42,43,44]. The perception of approximate tactical knowledge among players may lead to a greater understanding of tactical knowledge in a team and encourage cooperation [45,46,47,48]. Cooperation may result in a greater probability of achieving success through tactical and motor competence, with a focus on evaluating the effectiveness of all tactical actions [20,47]. Most studies focus on under (U) 17, U15, and U14 teams [46,48]. Only one study has evaluated tactical performance and motor effectiveness in U13, U15 and U17 teams [45,46]. Hence, there is limited understanding on the U11 competitive level according to player positions. Thus, the aim of this study is to verify the existence of significant differences in procedural knowledge between young football player positions in the U11, U13, and U15 level during a three-a-side SSG. It was hypothesized that there would be significant differences in procedural knowledge between different positions in young football players.

## 2. Materials and Methods

### 2.1. Participants

This was an observational and cross-sectional study considering 71 male football players with average age of 12.16 (±1.55) years. The players fit into the U11, U13 and U15 age categories. Among them, 11 were goalkeepers (GK), 22 were defenders, 15 were midfielders, and 23 were forwards. The U11 players trained twice per week (180 min/week), U13 players trained three times per week (270 min/week), and U15 players trained four times per week (360 min/week). The U11 and U13 teams participated in a regional championship and the U15 players competed in the National Portuguese Football Federation tournament. All the participants were amateur football players. For anthropometric evaluation, a digital scale (Tanita BC-545N^®^) and a digital stadiometer (SECA^®^ 242) were used [47]. The procedures were in accordance with the Helsinki Declaration on research with human beings. Written consent of the participants was provided. This research was approved by the scientific board of the Higher Institute of Educational Sciences of the ISCE Douro. Table 1 presents the number of players by position and total sample, considering mean and standard deviation of age, height, and body mass index. For inclusion, potential participants had to comply with the following inclusion criteria: (1) absence of injuries in the previous two months; (2) attendance of all training sessions during data collection; (3) participation in all playing time during the previous month of competition [49]; (4) sign informed consent forms to participate in this study. Those who did not comply with these inclusion criteria were excluded from participation in this study.

### 2.2. Procedure

The tactical procedural knowledge and tactical performance was assessed using the following instruments [33]: (i) Team Sports Performance Assessment Procedure (TSAP) [48]; (ii) Game Performance Assessment Instrument (GPAI) [50]; (iii) System of Tactical Assessment in football (FUT-SAT) [20]. The FUT-SAT evaluation was based on: (i) tactical principles; (ii) field location of the player’s actions; and (iii) tactical action outcomes [33]. Ten tactical principles were considered for analysis: (i) offensive: penetration, offensive coverage, depth mobility, space, and offensive unit; (ii) defensive: delay, defensive coverage, and balance [30,33]. Previous research reported intra- and inter-rater reliability values greater than k = 0.79 [30]. The TSAP assessed the performance of technical–tactical actions (specific skills) in their tactical contexts [48]. The GPAI is a multidimensional system created to assess game performance behaviors, players’ tactical comprehension, and players’ abilities to handle tactical challenges by choosing and putting to use the right set of skills [49,50]. Both tactical tools have a good construct validity in football [48,49,50]. Additionally, the motor effectiveness contextualized in tactical decision making was evaluated by an observational instrument developed by Quina et al. [33]. A high intra- and inter-observer reliability was reported for this observation tool in SSG, with average values of k = 0.89 and k = 0.80, respectively.

**Figure 1:**
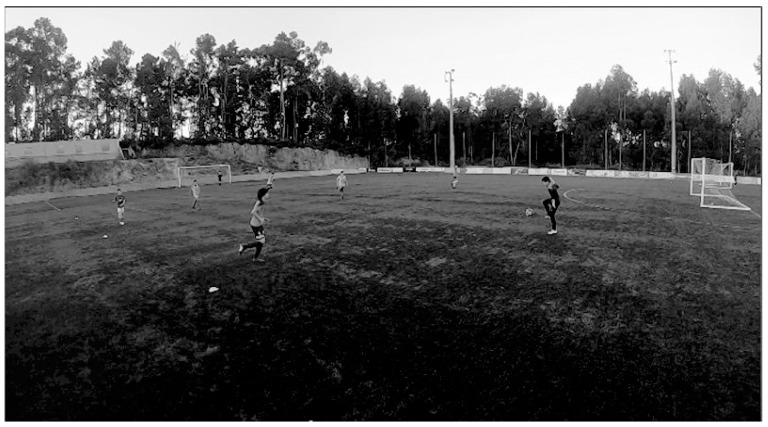
Four minutes of a SSG.

Current research also applied a SSG task design by 3 vs. 3 players (GK + 3 vs. 3 + GK) to assess the tactical performance; each player was evaluated according to the quality of their accomplishment of tactical actions, localization of the tactical action on the field, and action outcome. The games were videotaped and analyzed by three groups of observers. The instrument has a structure based on the principles of the game and integrates two dimensions (decision making and motor effectiveness), eight categories (the principles of the game), and performance indicators for each category. The decision making was evaluated in adequate/inadequate and the motor execution in effective/not effective. The goalkeepers were evaluated as field players and the games took place on a field of 36 m long and 27 m wide (see Figure 1). Lastly, each game had a duration of 4 min, which was considered the required amount of time to perform actions related to the game principles [20,48,49,50].

Validation procedures were conducted by two experts to analyze intra- and inter-observer reliability assessments. Inter-observer reliability values varied between 0.75 and 0.91 in the decision making dimension and between 0.73 and 0.84 in the motor difficulty dimension. The intra-observer reliability values were between 0.81 and 0.96 in the decision making dimension and between 0.78 and 0.95 in the motor intervention. SSGs were performed on natural pitches from 10:00 a.m. to 08:00 p.m. and with similar environment conditions (mean temperatures of 14.9 ± 5.3 °C). 

Players were randomly divided into groups of four. The players were asked to play according to the game’s rules. The U11 players played under the 7 vs. 7 official game rules in a SSG. The U13 and U15 teams played by the official 11 vs. 11 game rules in a SSG. A digital camera (GoPro^®^ version 2.01 Hero 6 Black) was used to record the game. The collected material was introduced into a computer (Apple^®^ MacBook Pro, Cupertino, CA, USA) for analysis. Video analyses were performed using LongoMatch^®^ software (LongoMatch^®^ version 1.5.9, Barcelona, Spain.) [50,51]. The instrument was based on a checklist with correct and incorrect actions. Based on the number of correct, incorrect, and total actions, it is possible to quantify the performance index by the ratio between the correct and the total actions [33]. The video analysis allowed the evaluation of the number of correct and incorrect actions for each player across moments [50,51]. The offensive and defensive principles of decision making are presented in Table 2 and Table 3, respectively. 

Motor effectiveness refers to the efficient execution of a motor task. This is related to the adequacy and choice of a technical action during a game situation [20]. The correct and incorrect classification was completed according to the assumptions presented in Table 4 and Table 5.

For each variable, successful actions to total actions ratios were calculated. The action indexes were calculated based on the correct and incorrect decisions for each variable previously described, so each player action on a given principle was pointed to as correct or incorrect. The indexes’ values ranges were between 0 and 1 [33]. Each index was calculated considering the following equation:

(1) $$I=\frac{No.\;of\;Right\;actions\;(n)}{No\;of\;incorrect\;actions\;(n)}$$

where *I* is the effectiveness index for each variable relating to motor efficacy and tactical actions. For the overall performance, calculation was made according to Equation (2):

(2) $$\mathrm{Performance}=\frac{\sum I+DMI+MEI}{10}$$

where DMI is the Decision-Making Index and MEI is the Motor Effectiveness Index. The calculation of the DMI index and MEI are presented in Equations (3) and (4), respectively:

(3) $$\mathrm{DMI}=\frac{\sum \mathrm{No}\;of\;correct\;actions\;for\;all\;variables}{\mathrm{No}\;of\;total\;actions\;for\;all\;the\;variables}$$

(4) $$\mathrm{MEI}=\frac{\sum \mathrm{No}\;of\;correct\;actions\;for\;all\;variables\;related\;to\;motor\;efficacy}{\mathrm{No}\;of\;total\;actions\;for\;all\;the\;variables\;related\;to\;motor\;efficacy}$$

### 2.3. Statistical Analysis

The Kolmogorov–Smirnov and the Levene test allowed the assessment of the sample for normality and homogeneity, respectively. Means and standard deviations were presented in descriptive analysis. Non-parametric tests were used for inferential analysis. The Kruskal–Wallis test was used to verify the existence of significant differences between field positions in the different principles of the game, motor effectiveness, and the overall performance. Effect sizes were calculated based on the H-statistic using eta square (η^2^). The interpretations of this effect size for non-parametric tests are as follows: *r* ≤ 0.1 = small effect size; 0.1 < *r* ≤ 0.3 = small-to-medium effect size; 0.3 < *r* ≤ 0.5 = medium-to-large effect size; and *r* > 0.5 = large effect size [52,53]. The statistical analysis was made with the IBM Statistical Package for Social Sciences version 22.0 (SPSS Inc., Chicago, IL, USA).

## 3. Results

Descriptive statistics for motor effectiveness, decision making for each game’s principles, and overall performance are presented in Table 6. The decision making indexes raged between 0.52 ± 0.29 and 0.76 ± 0.17 for goalkeepers, 0.58 ± 0.31 and 0.70 ± 0.24 for defenders, 0.50 ± 0.43 and 0.69 ± 0.32 for midfielders, and 0.50 ± 0.34 and 0.67 ± 0.16 for forwards. The decision making for each game’s principles indexes presented values between 0.48 ± 0.05 and 0.89 ± 0.18 for goalkeepers, 0.49 ± 0.04 and 0.86 ± 0.20 for defenders, 0.53 ± 0.09 to 0.90 ± 0.14 for midfielders, and 0.53 ± 0.08 and 0.85 ± 0.19 for forwards. The goalkeepers presented an overall performance of 0.66 ± 0.10, the defenders 0.63 ± 0.09, the midfielders 0.60 ± 0.12, and the forwards 0.59 ± 0.12.

The comparisons between positions for motor efficiency, decision making for each game’s principles, and overall performance are presented in Table 7. The group comparison presented significant differences in decision making for each game principle on the principle of contention (H = 7.920; *p* = 0.048, η^2^ = 0.267) between defenders and forwards (H = −11.92; *p* = 0.03) and defenders and midfielders (H = −16.13; *p* = 0.01). The defenders and forwards might have different behaviors to prevent the ball progression from the opponent with the ball; the same phenomenon might occur between defenders and midfielders. Thus, the way that the defensive team approached the offensive progression differs between the defenders and midfielders and defenders and forwards.

## 4. Discussion

The aim of this study was to evaluate tactical procedural knowledge in different field positions in U11, U13 and U15 young football players during a three-a-side SSG. It was hypothesized that there could be significant differences in procedural knowledge between different playing positions. The main result of the present study was that the decision making on the contention principle between defenders and forwards and defenders and midfielders was observed.

The tactical knowledge was assessed using procedural analysis. It is possible to assess procedural tactical knowledge using the FUT-SAT [30,41]. The FUT-SAT requires a game configuration of GR + 3 vs. 3 + GR, in a field with 36 m of length and 37 m wide, lasting four minutes. In the present study, the tactical procedural knowledge was evaluated using video recording and subsequent analysis, according to the observation instrument for the decision making for each game’s principal dimension and motor effectiveness based on previous literature [33,42]. The same data were analyzed according to offensive and defensive principles [2,48,49]. The results showed that tactical performance seems to vary between positions on different principles. This can be explained by the fact that, in the early years of sports practice, players are directed to the basic and general content of collective sports games [54,55]. During the game teaching process, it is possible to identify a tendency to direct it to the goal and forget defensive phase content, such as contention, coverage, balance, and concentration [56]. The process of sport initiation in collective games should provide the practitioner tactical procedural knowledge by activities related to the formal game [10,12,39]. That may help players to learn the offensive and defensive dynamics and processes of the sport [18,19,57]. 

The results of this study show that while there are no statistically significant differences in overall performance, there are some differences between playing positions. The overall performance that results from the mean of each decision making index and the motor effectiveness index was higher in goalkeepers compared to others. This can be explained by the transitions from 7 vs. 7 football to 11 vs. 11 [32,41], in which goalkeepers are subjected to more individualized training and, therefore, early specialization with the goal of goal defense. In addition, the orientation and directions given to goalkeepers do not vary significantly from the 7 to 11 football [58]. It should be considered that a complete football formation goes through the 3 vs. 3, 5 vs. 5, 7 vs. 7, 9 vs. 9 and finally 11 vs. 11 stages [41]. Such evolution leads to a growing number of actions both in number and complexity [59]. The number of interactions between goalkeepers and the remaining positions might be different [16,24,60]. The interactions between players may occur due the best player recognition and the best friend, i.e., young players often passed the ball to the best player or their best friend [29]. Base-playing with goalkeepers implied a need to play near the goal. Moreover, during training sessions, field players might be prone to play with the most successful offensive players [60]. Thus, the higher overall performance scores in goalkeepers might be explained by the reduced number of actions or the fear to fail during SSGs, as well as the need to prove themselves as good players [29]. Performance is given by the motor efficiency index and a correct action may result from a motor efficiency successful action with incorrect decision making [33]. The goalkeepers are prone to give more importance to better decision making to avoid conceding goals, instead of motor efficiency. Clemente et al. [61] verified that the offensive players and goalkeepers have a reduced number of interactions. Moreover, using the goalkeeper as an outfield player or as a forward player increases the probabilities of shots at goal and, thus, the probability of failure [61,62]. 

The practice quality and ability to play results from adequate decision making for each game principle, while motor effectiveness results from the actual moment of play [13,62]. Procedural tactical knowledge may still be influenced by training time [58]. Players exposed to longer training times and a larger number of training sessions tend to show regularly good performances [28,57]. In the current study, goalkeepers performed specific individualized work and trained with the collective team. On average, goalkeepers trained about 20 min per practice longer than the rest of the players, which may explain the best results achieved in overall performance. However, the years of practice of the evaluated subjects were not taken into account. The assimilation of tactical knowledge may vary from player to player and from position to position, as found in the present study. This can be explained by years of practice, complexity of practice, or individualization of actions for each position. A training methodology based on tactical dimensions may result in improvements to tactical behavior [30]. Upon that, players may experience situations similar to those of the real game [31]. A stratified and oriented training for each game principle, which maximizes the number of correct actions, may enhance tactical performance [17]. This can contribute to the development of game intelligence during the teaching and learning process, helping athletes to make the right choices during a given task [42]. However, there is insufficient research reporting similar analysis to that presented in the current study.

The decision making performance is related to several factors. Thus, the following limitations should be considered when interpreting the described results. Firstly, the sample is merely representative of a football club. Secondly, the number and hours of training were not equal between the studied competitive levels [63,64]. Thirdly, the expertise and years of practice of each player were not evaluated [65]. Fourthly, the cross-sectional design limits assumptions of causality and, thus, cannot predict performance over time and competitive levels [66]. Fifthly, neither the teaching–learning process nor the objectives of the training sessions were controlled [67,68]. Sixthly, cumulative effects were not controlled in this research [69,70]. Seventhly, maturational variables that can influence tactical behavior [28,71] and physical performance were not controlled [70]. Finally, the sample considered three different age categories (i.e., U11, U13 and U15), which have different maturational and cognitive abilities due to the stages of development [67,68,69]. Future studies should measure decision making principles with players of the same age category or by carrying out a comparison between them [70,71,72].

Tactical procedural knowledge is a favorable variable for tactical dimension control in football [20,49,50]. Moreover, it is a valid and reliable measure in children and young athletes [33]. The present study shows that tactical procedural knowledge may differ between playing positions. Moreover, the tactical principles of concentration and defensive unity were not evaluated in this 3 × 3 SSG format [30,31]. Future studies should consider the inclusion of these principles through a similar analysis in medium- and large-sided games (MSG, LSG) to assess tactical knowledge through the decision making and motor efficiency of young football players in these training task formats [28]. The evaluation and control of the actions may contribute to enhancement of individual and collective performance [28,55]. Future studies should: (i) assess player differences in performance with random and non-random divisions; (ii) compare players’ performances by competitive levels; (iii) perform a longitudinal analysis; (iv) compare tactical knowledge across teams. Based on the present results, coaches and analysts may better understand the tactical knowledge related to decision making and motor efficiency in each playing position [13]. Training sessions may then guide practitioners to improve their decision making based on science-based evidence. This may help players to understand what correct or incorrect actions are during a game.

## 5. Conclusions

This study concludes that the tactical process differed between positions in the contention principle. Thus, coaches, analysts, and players may better understand the possibility of performance variations between different positions. The transition of competitive levels and the evolution of the actions’ complexity may condition the specific performance.

## Figures and Tables

**Table 1:** Sample age, height, and body mass index by position.

Variables	Goalkeeper Mean (±SD)	Defender Mean (±SD)	Midfielders Mean (±SD)	Forwards Mean (±SD)	Total Mean (±SD)
Number of players	11	22	15	23	71
Age (year)	11.82 (±1.66)	12.14 (±1.55)	12.27 (±1.75)	12.30 (±1.49)	12.22 (±1.54)
Height	155.00 (±16.43)	154.82 (±12.03)	152.87(±15.25)	152.17 (±10.47)	153.10 (±12.21)
Body mass index	20.14 (±2.08)	19.70 (±2.46)	18.19 (± 2.89)	18.49 (±2.89)	18.50 (±0.26)

Notes: M = Mean; SD = Standard Deviation.

**Table 2:** Decision making classification regarding performance indicators as correct and incorrect in specific offensive game principles.

**Penetration**	Correct	When the player with ball chooses to direct the game towards the opposing goal when conditions to win spatial or numerical advantage are favorable.
	Incorrect	When the ball player chooses to move towards the opposing goal when conditions are clearly unfavorable.
**Offensive coverage**	Correct	It is correct when the attacker prefers to provide cover to the ball player when he is near him and the situation requires it.
	Incorrect	It is incorrect when the attacker prefers to cover the player with ball and he is already covered or not under pressure as the situation recommends another principle.
**Depth Mobility**	Correct	It is correct when the attacker chooses to position or move on/or to the half of the most offensive center of the game (H + OCG) with the intention of providing a forward pass option to the ball carrier when the situation allows.
	Incorrect	It is incorrect when the attacker chooses to position or move on/to the H + OCG with the intention of providing a pass line to the ball carrier when the situation recommends another principle.
**Space**	Correct	When the attacker (when appropriate) decides to stay away from the center of the game (CG) or move away from the CG to one of the side or bottom lines to extend the effective playing space (EPE) and create pass lines.
	Correct	When the attacker chooses to move from the periphery to the interior (or in the interior when appropriate) with the intention of creating or occupying space in a reduced pressure zone and creating pass opportunities.
	Correct	When the ball player chooses to play backwards when he does not have secure forward play options with the intention of creating spaces to perform other actions or to maintain possession of the ball.
	Incorrect	When the attacker decides to move towards the sideline or bottom line, when the ideal decision would be to approach the middle of the pitch.
	Incorrect	When the attacker chooses to move or position himself in the inherent play space when the situation requires another action to be taken.
	Incorrect	When the ball carrier chooses to play back when it is possible to play towards the opposing goal.

**Table 3:** Decision making classification regarding performance indicators as correct and incorrect in specific defensive game principles.

**Delay**	Correct	When the defender chooses to defend the player with the ball when he is undefended and nearby.
	Incorrect	When the defender chooses to defend the ball player when the ball is already under mark and the situation recommends another principle.
**Defensive Coverage**	Correct	When the defender chooses to cover the remaining defender when he is near him and the situation demands it.
	Incorrect	When the defender chooses to cover the defender when the situation requires another principle.
**Balance**	Correct	When the defender chooses to keep up (when necessary) with the attackers and prevents them from receiving the ball in favorable situations.
	Incorrect	When the defender chooses to keep up the attackers in mobility when the situation requires another principle.
**Concentration**	Correct	When the defender is not restrained by the attackers or covering, he chooses to position himself between the H + OCG and his goal.
	Incorrect	When the defender chooses to position himself in the defensive containment zone when the action requires another principle.

**Table 4:** Classification of motor effectiveness regarding performance indicators as correct and incorrect for specific offensive game principles.

**Penetration**	Correct	When the shot results in a goal.
	Correct	When the action of the ball player results in the maintenance of the ball possession and numerical or spatial advantage.
	Incorrect	When the ball player loses possession of the ball.
	Incorrect	When the ball player throws or drives the ball into a high density zone.
**Principle Offensive coverage**	Correct	When the attacker’s movement/positioning ensures a safe pass opportunity and allows containment in the event of the ball carrier being disarmed.
	Incorrect	When the attacker’s movement/positioning does not guarantee a pass opportunity or does not allow to defend in case of disarming.
**Mobility Principle**	Correct	When the attacker’s movement creates the possibility of passing forward.
	Correct	When the attacker’s movement creates spaces in the H + OCG
	Incorrect	When the attacker’s movement does not create possibility of passing forward.
	Incorrect	When the attacker’s movement does not create spaces in the H + OCG.
**Space**	Correct	When the attacker’s movement/positioning creates a pass opportunity to the front of the ball line.
	Correct	When attacking movement allows the player to position himself within the lowest pressure EPE and create a pass opportunity.
	Correct	When the ball carrier plays towards his goal, he seeks to create conditions for himself or his teammate to progress, shoot, pass, or retain the ball possession.
	Incorrect	When moving/positioning does not increase EPE or does not provide a pass opportunity.
	Incorrect	When the attacker’s movement does not allow him to position himself in a lower pressure zone within the EPE or create a pass opportunity.
	Incorrect	When the ball player loses the ball trying to play towards his goal.

**Table 5:** Classification of motor effectiveness regarding performance indicators as correct and incorrect for specific defensive game principles.

**Delay**	Correct	When the defender recovers the ball by interception or tackling.
	Correct	When the defender’s action interferes with the attacker’s action, leading to a less dangerous action.
	Incorrect	When the defender’s action does not condition the attacker’s action.
**Defensive Coverage**	Correct	When the defender stands in the most H + OCG between the contention defender and the goal to cut forward pass opportunities or, in case the holding defender is overtaken, prevent an attack.
	Incorrect	When the position of the defender does not allow him to cut forward pass opportunities or, if the defending defender is overtaken, an attack commences.
**Balance principle**	Correct	When the defender’s movement/positioning allows the player to stymie the offensive mobility of the game creator and defend opponents who may receive the ball or intercept pass opportunities.
	Incorrect	When the defender’s movement/positioning does not allow defensive stability to be created in H + OCG or adjacent zones.
**Concentration**	Correct	When the movement/positioning of the defender contributes to conditioning the attacking opponent in high-risk zones.
	Incorrect	When the movement/positioning of the defender does not contribute to conditioning the attack on high-risk zones.

**Table 6:** Mean and standard deviation of motor effectiveness, decision making for each game’s principle, and overall performance by field positions.

Variables	Goalkeeper (*n* = 11)	Defenses (*n* = 22)	Midfielders (*n* = 15)	Forwards (*n* = 23)	Total (*n* = 23)
Motor efficiency	Mean ± SD	Mean ± SD	Mean ± SD	Mean ± SD	Mean ± SD
Motor efficiency index	0.67 ± 0.11	0.64 ± 0.10	0.62 ± 0.12	0.61 ± 0.13	0.61 ± 0.13
Penetration	0.52 ± 0.29	0.58 ± 0.31	0.51 ± 0.35	0.58 ± 0.34	0.58 ± 0.34
Offensive coverage	0.64 ± 0.32	0.65 ± 0.21	0.69 ± 0.32	0.61 ± 0.22	0.61 ± 0.22
Mobility	0.61 ± 0.19	0.69 ± 0.18	0.65 ± 0.19	0.62 ± 0.17	0.62 ± 0.17
Space	0.72 ± 0.21	0.69 ± 0.20	0.65 ± 0.17	0.67 ± 0.16	0.67 ± 0.16
Containment	0.76 ± 0.14	0.63 ± 0.28	0.65 ± 0.19	0.61 ± 0.30	0.61 ± 0.30
Defensive coverage	0.76 ± 0.16	0.63 ± 0.20	0.61 ± 0.19	0.60 ± 0.29	0.60 ± 0.29
Balance	0.76 ± 0.17	0.70 ± 0.24	0.66 ± 0.26	0.57 ± 0.23	0.57 ± 0.23
Concentration	0.66 ± 0.27	0.59 ± 0.25	0.50 ± 0.43	0.50 ± 0.34	0.50 ± 0.34
Decision making	0.48 ± 0.05	0.49 ± 0.04	0.53 ± 0.09	0.53 ± 0.08	0.53 ± 0.08
Penetration	0.65 ± 0.33	0.65 ± 0.34	0.71 ± 0.27	0.63 ± 0.40	0.63 ± 0.40
Offensive coverage	0.85 ± 0.18	0.86 ± 0.20	0.79 ± 0.24	0.73 ± 0.34	0.73 ± 0.34
Mobility	0.61 ± 0.28	0.64 ± 0.32	0.72 ± 0.28	0.74 ± 0.29	0.74 ± 0.29
Space	0.69 ± 0.22	0.71 ± 0.19	0.71 ± 0.16	0.68 ± 0.22	0.68 ± 0.22
Containment	0.86 ± 0.15	0.67 ± 0.31	0.90 ± 0.14	0.85 ± 0.19	0.85 ± 0.19
Defensive coverage	0.80 ± 0.25	0.70 ± 0.24	0.69 ± 0.27	0.67 ± 0.30	0.67 ± 0.30
Balance	0.73 ± 0.27	0.62 ± 0.37	0.66 ± 0.30	0.54 ± 0.31	0.54 ± 0.31
Concentration	0.89 ± 0.18	0.68 ± 0.27	0.66 ± 0.32	0.67 ± 0.26	0.67 ± 0.26
Overall performance	0.66 ± 0.10	0.63 ± 0.09	0.60 ± 0.12	0.59 ± 0.12	0.59 ± 0.12

Notes: SD = Standard Deviation; n = sample size.

**Table 7:** Mean comparisons between ranks in motor efficiency and decision making for each game principle.

Variables	Comparison between Groups		
	H	*p*	η^2^
Motor efficiency			
Motor efficiency index	2.175	0.540	0.384
Penetration	0.718	0.869	0.414
Offensive coverage	1.205	0.752	0.404
Mobility	2.139	0.544	0.385
Space	0.368	0.947	0.421
Containment	3.368	0.338	0.360
Defensive coverage	4.521	0.210	0.336
Balance	5.430	0.143	0.318
Decision making for each game principle			
Concentration	1.920	0.589	0.389
Decision making	5.852	0.119	0.309
Penetration	0.150	0.985	0.426
Offensive coverage	1.886	0.596	0.390
Mobility	2.677	0.444	0.374
Space	0.118	0.990	0.426
Containment	7.922	0.048 *	0.267
Defensive coverage	1.858	0.602	0.391
Balance	2.747	0.432	0.373
Concentration	5.998	0.112	0.306
Overall performance	2.455	0.484	2.455

Notes: η^2^—partial eta squared; H = H-statistics. * Statistical significance for *p* < 0.05.

## Data Availability

Data are available upon request to the contact author.
